# A Review of Methods to Modify the PDMS Surface Wettability and Their Applications

**DOI:** 10.3390/mi15060670

**Published:** 2024-05-21

**Authors:** Lucas B. Neves, Inês S. Afonso, Glauco Nobrega, Luiz G. Barbosa, Rui A. Lima, João E. Ribeiro

**Affiliations:** 1 Instituto Politécnico de Bragança, Campus Santa Apolónia, 5300-253 Bragança, Portugal; neves.lucas17@gmail.com; 2 Instituto Federal de Educação, Ciência e Tecnologia do Rio Grande do Sul (IFRS), Campus Erechim, Erechim 99713-028, RS, Brazil; luiz.barbosa@erechim.ifrs.edu.br; 3 MEtRICs, Mechanical Engineering Department, University of Minho, Campus de Azurém, 4800-058 Guimarães, Portugal; inesafonso@ipb.pt (I.S.A.); glaucotvn@hotmail.com (G.N.); rl@dem.uminho.pt (R.A.L.); 4 CIMO, Instituto Politécnico de Bragança, Campus S. Apolónia, 5300-253 Bragança, Portugal; 5 CEFT—Transport Phenomena Research Center, Faculty of Engineering, University of Porto, Rua Dr. Roberto Frias, 4200-465 Porto, Portugal; 6 Associate Laboratory in Chemical Engineering (ALiCE), Faculty of Engineering, University of Porto, 4200-465 Porto, Portugal

**Keywords:** polydimethylsiloxane (PDMS), wettability modification, surface treatment, nanomaterial incorporation, PDMS applications

## Abstract

Polydimethylsiloxane (PDMS) has attracted great attention in various fields due to its excellent properties, but its inherent hydrophobicity presents challenges in many applications that require controlled wettability. The purpose of this review is to provide a comprehensive overview of some key strategies for modifying the wettability of PDMS surfaces by providing the main traditional methods for this modification and the results of altering the contact angle and other characteristics associated with this property. Four main technologies are discussed, namely, oxygen plasma treatment, surfactant addition, UV-ozone treatment, and the incorporation of nanomaterials, as these traditional methods are commonly selected due to the greater availability of information, their lower complexity compared to the new techniques, and the lower cost associated with them. Oxygen plasma treatment is a widely used method for improving the hydrophilicity of PDMS surfaces by introducing polar functional groups through oxidation reactions. The addition of surfactants provides a versatile method for altering the wettability of PDMS, where the selection and concentration of the surfactant play an important role in achieving the desired surface properties. UV-ozone treatment is an effective method for increasing the surface energy of PDMS, inducing oxidation, and generating hydrophilic functional groups. Furthermore, the incorporation of nanomaterials into PDMS matrices represents a promising route for modifying wettability, providing adjustable surface properties through controlled dispersion and interfacial interactions. The synergistic effect of nanomaterials, such as nanoparticles and nanotubes, helps to improve wetting behaviour and surface energy. The present review discusses recent advances of each technique and highlights their underlying mechanisms, advantages, and limitations. Additionally, promising trends and future prospects for surface modification of PDMS are discussed, and the importance of tailoring wettability for applications ranging from microfluidics to biomedical devices is highlighted. Traditional methods are often chosen to modify the wettability of the PDMS surface because they have more information available in the literature, are less complex than new techniques, and are also less expensive.

## 1. Introduction

The silicone elastomer known as polydimethylsiloxane (PDMS) has been widely employed as the main substrate in various micro/nano applications. These include microcontact printing [1], microfluidics [2,3,4,5], and microreactors [6], as well as in fabric coatings and water–oil separation [7,8,9,10,11,12], among many other applications. This is due to its attractive characteristics, such as easiness of manufacturing, non-toxicity, optical transparency, low cost, favourable biocompatibility, elastic nature, ease to process and advantageous chemical and physical properties, the malleability for moulding on (sub)micrometric scales, and effective adhesion to itself and to the glass [13,14,15,16,17]. However, to improve the surface properties of PDMS, such as wettability, it is essential to modify the material to specific conditions.

**Figure 1:**
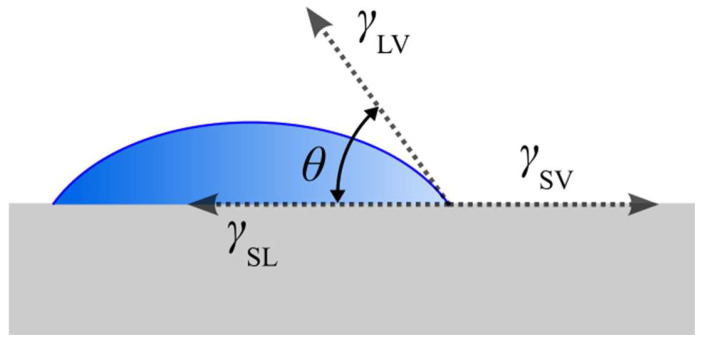
Contact angle (*θ*) and interfacial tensions (γ) between the solid (S), liquid (L), and vapour (V).

Wettability, defined by the contact angle (*θ*), refers to the measure of adhesion between a liquid and a solid surface, which performs a fundamental role in understanding the interactions between materials and liquids. The hydrophobicity or hydrophilicity of a surface is defined by the surface energy of the material. For PDMS, hydrophobicity is given by the presence of methyl groups (-CH_3_) in its structure, which gives it low polarity and weak interactions with water molecules, making it a hydrophobic surface. The presence of hydroxyl groups (-OH) or other polar groups increases hydrophilicity, providing a stronger interaction with water and making the surface more hydrophilic [18,19]. The contact angle lesser than 10° characterises the surface as superhydrophilic; between 10° ≤ *θ* < 90° as hydrophilic, favouring the spread of the liquid; from 90° ≤ *θ* < 150° is considered hydrophobic; and greater than 150° defines the surface as superhydrophobic, where the droplet tends to take on a more spherical shape [20,21,22,23,24]. The basic model for the contact angle of an idealised solid surface is described by the Young equation, Equation (1) [25]:

(1) $$cos\left(\theta \right)=\frac{\gamma_{SV}-\gamma_{SL}}{\gamma_{LV}}$$

where *θ* is the contact angle, $\gamma_{SV}$ is the interfacial tensions between solid and vapour, $\gamma_{SL}$ is the interfacial tensions between solid and liquid, and $\gamma_{LV}$ is the interfacial tensions between liquid and vapour. The relation can be better visualised in Figure 1.

As mentioned, the Young equation is nonetheless based on an ideal surface without roughness. However, when roughness is considered, the contact angle is corrected by a roughness factor. The Wezel Theory [26] for wettability on a rough surface is demonstrated in Equation (2):

(2) cos⁡θ*=rcos⁡θ

where θ* is the apparent contact angle and *r* is the roughness factor calculated as the ratio between the ideal and real surface. The equations show that an increase in roughness makes the hydrophilic surface more hydrophilic and the hydrophobic surface more hydrophobic.

The roughness, meanwhile, has only a fractional impact on the contact angle, as well as on parameters such as porosity and wettability of the material. Other models, such as the Cassie–Baxter model (Equation (3)), can be applied specifically to hydrophobic surfaces with sharp edges, for example [26,27].

(3) cos⁡θ*=f−1+rfcos⁡θ

where f is the projected wet area, and r represents the roughness factor.

Superhydrophobic or superhydrophilic characteristics can be applied in various areas. Examples of the use of superhydrophobic surfaces include self-cleaning paint and acrylics that prevent the adhesion of dirt, improving the surface’s capacity to be cleaned by rain. These coatings can also be applied to avoid snow accumulation on tall structures such as antennas; in toilet urinals to repel urine, reducing the need for water; in paints for architectural heritage; in photovoltaic panels to remove dust and increase efficiency; in functional textiles with the use of nanoparticles; and in other diverse applications as anti-icing properties in the aerospace industry and wind blades beyond anti-fouling purpose [28,29,30,31,32]. Superhydrophilic applications include self-cleaning paint and coatings that expedite the cleaning process with a stream of water or rain. Antifogging mirrors, glasses, and shields are useful for preventing fogging caused by humidity. Additionally, antifogging bags and packaging films are employed to increase the water capacity to extend [33]. Other applications in heat transfer may also be desirable: in a two-phase heat transfer device, a superhydrophilic surface improves the ability of water to wet the hot area where the evaporation occurs, while a superhydrophobic surface is effective in repelling droplets on the cold surface formed by the condensation of vapour [29].

Despite the great advantages as anti-corrosion, self-cleaning, and anti-icing [34] due to the inherently hydrophobic characteristics, PDMS imposes limitations for certain purposes. For example, for applications that require contact angles between 20 and 70°, such as liquid lenses, it is essential to reduce the hydrophobicity of PDMS [35]. In addition, there is a limitation in the use of PDMS as a biomaterial and cell adhesion on cell culture substrates [36] because of its hydrophobic nature. There is also a difficulty of specific equipment and time-consuming procedures, obstacles that can be manifested in surface hydrophilisation [28,37,38].

A wide range of papers were found on PDMS and its properties, specifically its wettability and how to modify it, mainly by traditional methods. These traditional methods are often chosen to change the wettability of the PDMS surface because they have more information available in the literature, are less complex than new techniques, and are less expensive. However, no research was found that objectively explained the main traditional methods of altering the wettability of the PDMS surface, the results of altering the contact angle, and other characteristics associated with this property.

Therefore, this article presents a brief review of the main treatments and their studies to change the wettability of the PDMS surface, detailing the phenomena involved in the interaction between the surface and the fluid, the advantages and disadvantages of using this type of method, and the main results achieved as well as their main applications. It also approaches the roughness, additives, changes in the molecular surface, and other parameters that have influence in the contact angle. The main observations on the methods and future prospects in the area are also addressed.

Table 1 presents a summary of some advantages and disadvantages of the main methods used for the fabrication of PDMS-based superhydrophilic/superhydrophobic coatings, as well as their major applications and water contact angle (WCA) changes over time. The methods considered most important are further discussed.

## 2. Oxygen Plasma Treatment

**Figure 2:**
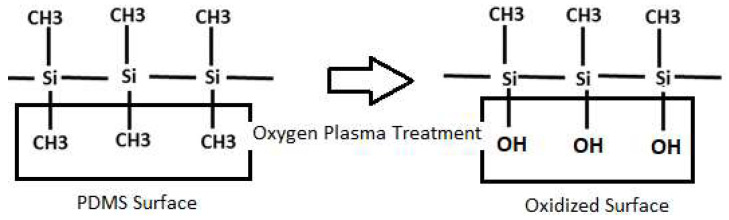
Surface reaction of PDMS under O_2_ plasma treatment.

The PDMS surface modification through oxygen plasma treatment stands out as a fundamental approach, particularly in the fast prototyping of PDMS microfluidic devices [47]. This process involves the introduction of polar functional groups, notably the silanol group (SiOH), through oxidation processes and the subsequent removal of hydrocarbon groups by surface corrosion [35,48], as demonstrated in Figure 2. These functional groups alter the surface characteristics of PDMS, converting it from hydrophobic to hydrophilic. However, it is important to note that, on one hand, plasma-treated surfaces exhibit a recovery of hydrophobicity within minutes of exposure [49], particularly when subjected to bonding and heat treatment [50]. On the other hand, prolonged plasma treatments can cause undesirable surface cracks that compromise the integrity of the device [48,51,52].

The hydrophobic recovery of PDMS has been extensively investigated using techniques such as measuring the WCA on the surface [53], scanning electron microscopy (SEM) [47], and X-ray photoelectron spectroscopy (XPS) [54]. Several factors play crucial roles in this process, including the reorientation of polar groups from the surface to the interior of the material, the diffusion of low molecular weight species from the bulk to the surface, and the condensation of hydroxyl groups [55]. In addition, the recovery rate is influenced by storage conditions such as temperature, humidity, and the presence of aqueous fluids and surfactants used to preserve PDMS devices [39,56,57,58,59,60,61]. Recently, several studies have been carried out an analysis of each of these parameters to alter the surface properties of PDMS, as is shown below.

However, such techniques may present some challenges, including sophisticated and time-consuming mechanisms, expensive demands for vacuum apparatus and DC power supply for high voltage generation, safety issues, and well-trained technicians [38]. Also, this technique suffers from a short lifetime before hydrophobic recovery [48].

### 2.1. Principles of Oxygen Plasma Treatment and Applications of PDMS Treated with Oxygen Plasma

In oxygen plasma treatment, the oxygen is ionised by the application of high voltage to form ions and free electrons. These reactive species, such as oxygen ions, play a key role in their interactions with material surfaces, facilitating chemical and physical attacks. This process results in the breaking of chain bonds in the PDMS molecular structure, thus introducing oxygen-containing functional groups. In addition, oxygen plasma cleans surfaces, removes contaminants, and increases surface energy, facilitating the adhesion of coatings [62]. Precise control of the process parameters, such as exposure time, power, and gas composition, are essential to adapt the surface properties to the specific needs of the application [63].

The oxygen plasma chamber has a controlled environment which generally consists of a vacuum chamber equipped with electrodes. Oxygen gas is put into the chamber, and then an energy source, such as a high-frequency current, is applied to the electrodes, ionising the gas and thus generating a reactive plasma. This plasma interacts with the PDMS surface, causing chemical and physical changes. The structure of the chamber and the operating parameters, such as pressure, plasma power, and treatment time, are adjusted according to the application and to optimise the effectiveness of the modification [49]. In a study [58], this experiment was conducted on microchannels using an oxygen plasma reactor (Series 790, Plasma-Therm, Inc., St. Petersburg, FL, USA), and the results showed an improvement in the hydrophilicity of the PDMS surface, reducing the WCA from 120° to 17° with a 300 s treatment.

In oxygen plasma treatment for polydimethylsiloxane modification, power, time, speed, and oxygen concentration are the main factors. Increasing power can exacerbate surface attacks, but balance is key to avoiding damage in the PDMS. Exposure time affects the introduction of modification and needs to be optimised to achieve the desired properties without compromising the integrity of the material. Exposure speed affects the uniformity of the retouching and requires precise control. Oxygen concentration determines the introduction of functional groups but needs to be balanced to avoid excessive degradation [64]. Careful adjustment of these parameters is crucial to obtaining the desired results when modifying the surface of PDMS. This technique is widely used to modify surfaces due to its ability to act on surface layers without deeply affecting the underlying structure. In addition, plasma is effective in sterilisation and in the chemical and morphological modification of surfaces [65,66].

The oxygen plasma treatment technique plays a crucial role in several areas due to its ability to alter the surface properties of materials. This approach finds application in a wide range of sectors, including microfluidic devices [51], adhesives and sealants [67], microfabrication of electronic circuits [68], biomedical implants [69], packaging coatings [70], biomaterials [40], and chemical sensors [71], as well as in the manufacture of lenses and optics [69]. Oxygen plasma treatment is preferred in all these applications due to its ability to characterise surface properties, such as adhesion, wettability, and functionality, according to the specific needs of each sector.

### 2.2. Effects of Oxygen Plasma Treatment on PDMS

Typically, the changes resulting from plasma exposure depend on variables such as applied power, exposure time, and the type of precursor gas, directly affecting the properties of the treated surface [72]. Plasma technology offers flexibility and desirable cellular responses, making it a commercially viable option [73,74,75]. The hydrophilicity resulting from the formation of an oxide layer on the surface during exposure to plasma tends to decrease gradually over time, due to the migration of oligomers, molecules composed of a small number of monomers, which are the basic units of a macromolecule, from the interior to the surface and the reorientation of polar groups [49,58,76].

**Figure 3:**
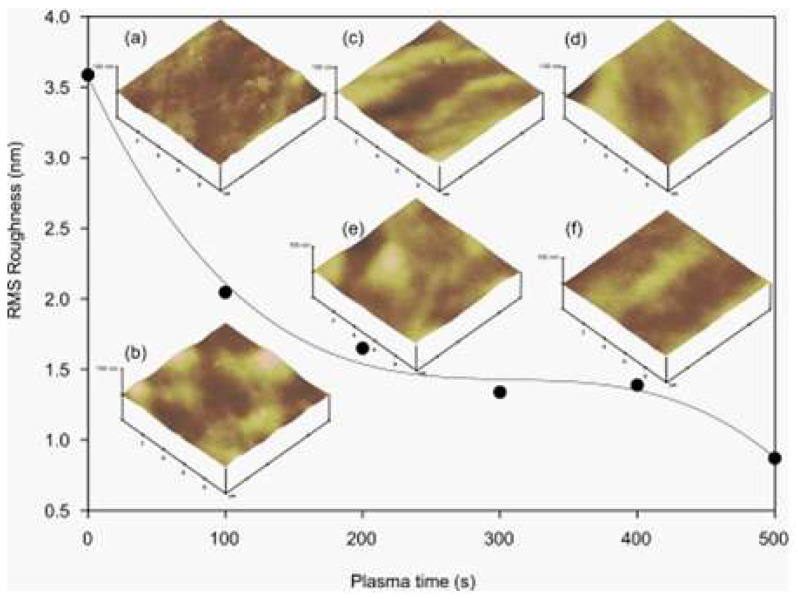
Mean square roughness as a function of oxygen plasma treatment time. The respective AFM images are taken from the sample with oxygen plasma exposure of (**a**) 0 s, (**b**) 100 s, (**c**) 200 s, (**d**) 300 s, (**e**) 400 s, and (**f**) 500 s. Obtained from [58].

In a study conducted by Tan et al. [58], a simple protocol was developed to produce hydrophilic and usable PDMS microchannel devices. This protocol involves a second prolonged oxygen plasma treatment and proper storage of the devices. The results indicated that, under a plasma power of 70 W, prolonged treatment of more than 5 min resulted in a PDMS surface maintaining the hydrophilicity for more than 6 h. In addition, storing the treated devices in deionised water allowed them to maintain their hydrophilicity for weeks. Analysis using atomic force microscopy (AFM) revealed that a longer exposure time to the oxygen plasma resulted in a smoother surface. The study used six different treatment times, ranging from 100 to 500 s, while the plasma power remained constant at 70 W. After plasma treatment, the devices were immersed in deionised water to remove air bubbles and stored in a vacuum chamber for seven days. AFM analyses were conducted within one hour of exposure to oxygen plasma. The roughness analysis revealed that the oxygen plasma treatment significantly reduced the roughness of the PDMS surface. For example, the root mean square (RMS) roughness dropped from 3.6 nm to 0.9 nm after a 500 s treatment, as shown in Figure 3. In addition, the formation of nano-cracks during oxygen plasma treatment proved potentially beneficial, as recent studies suggest that these nano-cracks can be furnished with adhesive proteins, which aids in the growth and modulation of biological cells [77].

**Figure 4:**
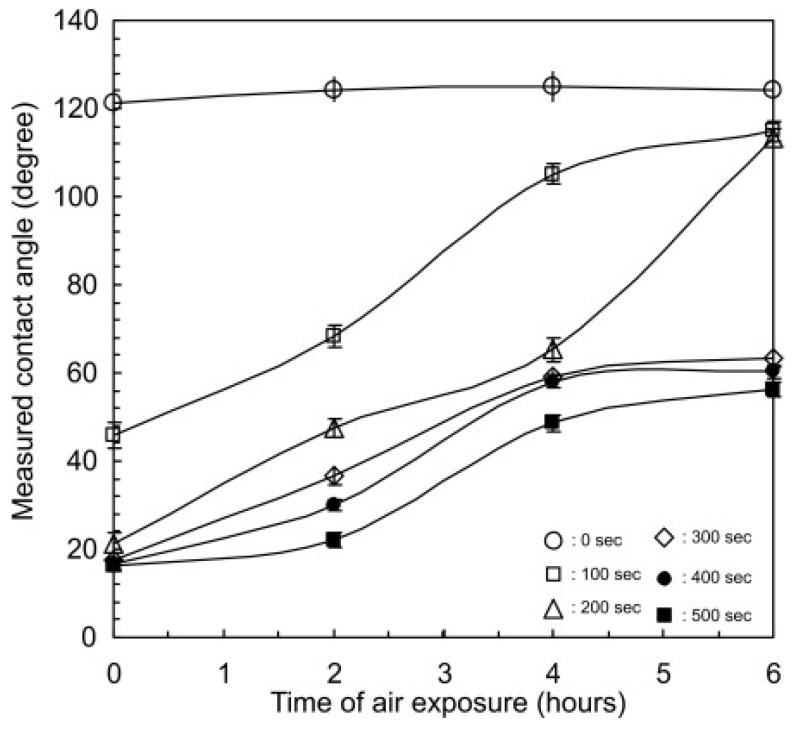
Water contact angle (WCA) as a function of air exposure time. Obtained from [58].

Figure 4 illustrates how the WCA varies over the air exposure time and, in general terms, how a longer plasma treatment reduces it. Untreated PDMS maintained a WCA of around 120°. After a 100 s treatment, the WCA dropped to 46° and then returned to around 115° after 6 h. With 200 s of treatment, the WCA dropped even further, reaching 21°, and then approached 115° again after 6 h. For treatments longer than 300 s, the initial WCA was 17°, and after 6 h, it remained between 50° and 60°. This highlights how the plasma treatment time has a notable impact on the wettability properties of the PDMS surface [58].

The results indicate that storing PDMS samples in water and under a vacuum can extend the hydrophilicity for at least 7 days, due to the surface energy of the water, preventing the rearrangement of the silanol group (SiOH) on the channel walls. This suggests that the surface energy of the fluid can affect the storage time of hydrophilic PDMS devices. Furthermore, it should be considered that the analysis of the surface in channels cannot be directly compared to that of a flat surface due to the influence of channel confinement.

**Figure 5:**
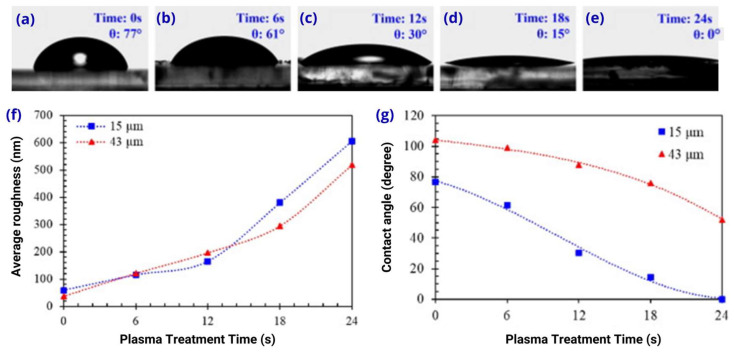
The contact angle between the glycerol drop and the PDMS film with a thickness of 15 ± 1.17 μm without oxygen plasma treatment (**a**) and with oxygen plasma treatment times of 6 s (**b**), 12 s (**c**), 18 s (**d**), and 24 s (**e**). Average roughness of PDMS thin films at thicknesses of 43 ± 1.44 µm and 15 ± 1.17 µm (**f**). Glycerol contact angles on PDMS films with different plasma treatment times (**g**). Adapted from [35].

Duangkanya et al. [35] presented the dependence of the oxygen plasma treatment time on the hydrophilicity on the surface of different thicknesses of PDMS thin films. For a thickness of 43 ± 1.44 µm, the initial contact angle (CA) between glycerol and the untreated PDMS film was around 104°; as the film was treated with oxygen plasma, it decreased to 52° at a surface treatment time of 24 s. As can be seen in Figure 5a–e for a thickness of 15 ± 1.17 µm, the initial untreated angle was 77°, and, for the process, it decreased to 15° when the surface treatment time was 18 s. This shows the influence of the surface treatment on the CA, and it was also noted that the rate of hydrophilic transformation of the thin film was much faster than that of the thick PDMS film.

In the same work mentioned above, the PDMS films with a thickness of 43 ± 1.44 µm, without the oxygen plasma treatment time, had an average surface roughness of 38 nm and gradually increased to 519 nm at the oxygen plasma treatment time of 24 s (Figure 5f), contrary to Tan et al. [58] Similarly, PDMS films with a thickness of 15 ± 1.17 µm, without the oxygen plasma treatment time, had an average surface roughness of 59 nm and increased exponentially to 606 nm at the 24 s oxygen plasma treatment time.

The CA, in general, is inversely related to the roughness of the film, especially when it comes to hydrophilic surfaces, where the apparent CA is less than 90° [78]. Figure 5g illustrates how the CA decreased more slowly for thick PDMS films during a 24 s plasma treatment period. For thinner films, this decrease occurs more quickly.

An innovative approach to surface modification with plasma [74], employed a scanning radical microjet (SRMJ) with a microplasma of *O*_2_ to control the adhesion of biological cells. The used technique has several advantages, such as higher rates of surface modification while minimising damage compared to conventional plasma exposure. Furthermore, the additional scanning speeds contribute to improving the hydrophilicity of the surface and significantly reducing roughness. However, in agreement with Duangkanya et al. [35], the average roughness values (*Ra*) and root mean square (*R*MS) show a gradual increase in their values as oxygen flow rates increase [74].

**Figure 6:**
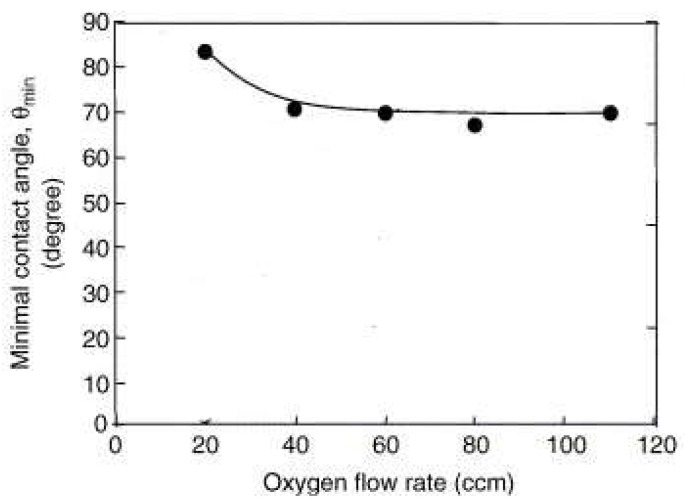
Relationship between minimum contact angle, θ min, and width of treated line, W, for different oxygen flow rates. Adapted from [74].

Figure 6 displays the effects of oxygen flow rates on WCA on PDMS surfaces treated by SRMJ. As the oxygen flow rate increased from 20 to 40 ccm, there was a gradual reduction in the minimum angle (θ min) from 83° to around 69°. However, as the oxygen flow rate continued to increase, from 40 ccm to 110 ccm, the hydrophilicity of the PDMS surface stabilised, resulting in a practically constant WCA.

The AFM observation results in this study demonstrated that biological cells attach more easily when the surface roughness values are higher. Tan et al. [74] also stated that although the result can be attributed to the presence of stronger cell–surface interactions on rough surfaces, it is essential to conduct further studies to clarify the cause-and-effect relationship between surface roughness and cell adhesion at the nanometric level.

Amerian et al. [38] exposed PDMS samples to plasma in a cylindrical reactor containing *O*_2_ gas with variable plasma exposure times of 0.5 min, 2.5 min, and 5 min for analysis, keeping the other factors constant. The WCA of the PDMS strips for samples with only 30 s of plasma exposure decreased from 117.9° to around 101.17°, then changed to 87.18° after 2.5 min, and after 5 min, the WCA dropped to 40°.

**Figure 7:**
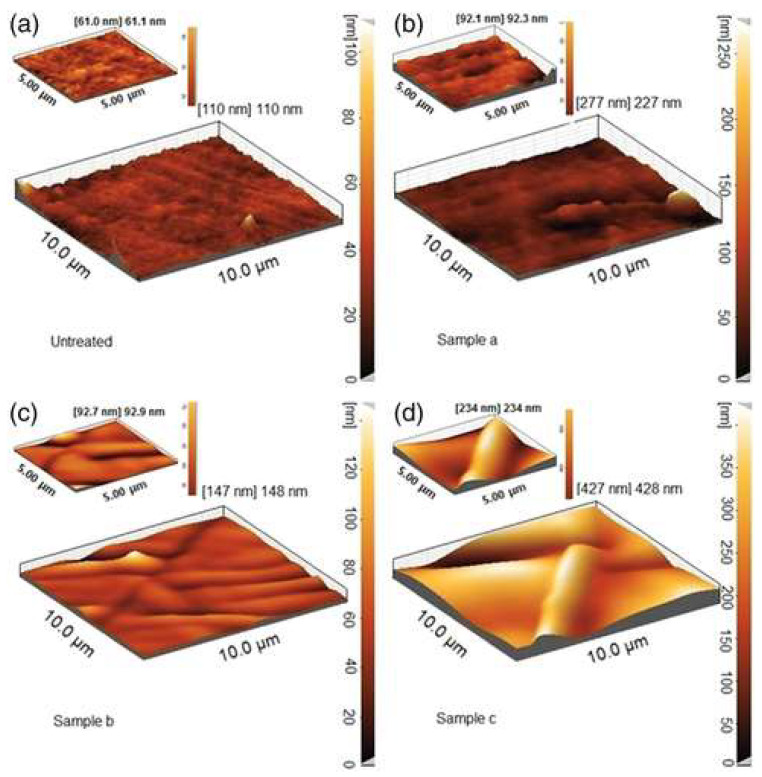
AFM images: (**a**) untreated, (**b**) sample a (0.5 min), (**c**) sample b (2.5 min), and (**d**) sample c (5 min). AFM, atomic force microscopy. Adapted from [38].

The results of the study revealed that the roughness of the PDMS surface increases with the plasma exposure time interval, according to Figure 7. The authors also reported that the PDMS films used in this study tended to crack after long plasma exposure times.

## 3. UV-Ozone Treatment

In this section, UV-ozone treatment in order to modify the surface energy and incorporate polar groups is discussed, since UV irradiation in combination with ozone has been widely employed for these purposes [41,79,80,81].

According to Berdichevsky et al., the combination of UV light and ozone proved to be effective in modifying the wettability of PDMS [42], with results comparable to oxygen plasma treatment, but at a slower rate [82], allowing for a precise control of wettability. For example, the oxygen plasma process of 400 W at 10 mTorr for 1 min resulted in a WCA of less than 10° [83], while UV ozone treatment required approximately 1 h to achieve a similar WCA, e.g., [82,84].

Polydimethylsiloxane, when subjected to UV-ozone treatment, results in the oxidation of the chains on the surface in more internal layers in the PDMS, free of cracks, in contrast to the approach using oxygen plasma [85,86]. One study described the use of UV/ozone treatment in the production of microfluidic devices [42]. In particular, they explored the mass conversion of PDMS by means of deep penetration, as well as the complete oxidation of thick membranes by means of UV-ozone treatment [43,87].

**Figure 8:**
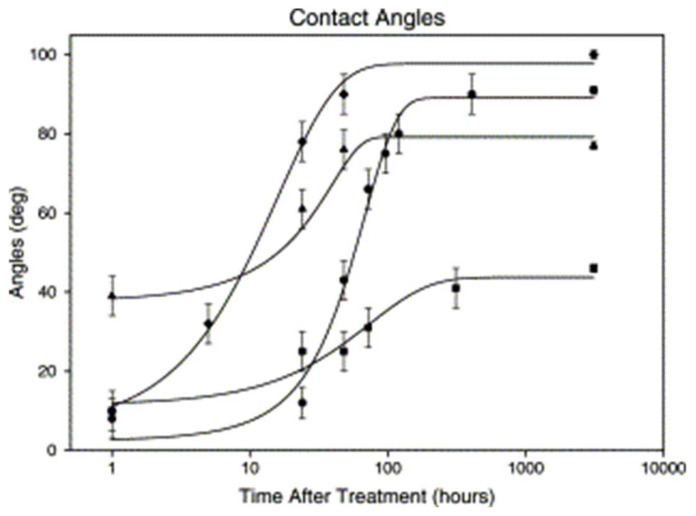
Contact angles versus time (logarithmic scale). Symbols: (•) PDMS membrane treated with UV-ozone for 60 min of 14 μm; (⧫) bulk PDMS treated with RF oxygen plasma for 1 min; (▴) treated with UV-ozone for 30 min the bulk PDMS; and (■) 120 min UV-ozone exposure of the 14 μm PDMS membrane. Obtained from [42].

The restoration of hydrophobicity in this study was noted on surfaces treated with RF oxygen plasma and bulk PDMS subjected to UV-ozone over 1 to 2 days. Similarly, the membrane treated for 60 min exhibited an analogous behaviour, while the membrane treated for 120 min, according to the authors, remained hydrophilic for more than 3 months, although Figure 8 only shows experimental points of approximately up to 21 days. The discrepancy in the results is attributed to the hydrophobic recovery mechanism, indicating the significant influence of the diffusion of low molecular weight PDMS chains. The rapid recovery after UV-ozone treatment is explained by the high diffusivity of the PDMS chains in the oxidised layer, while the slower recovery after UV-ozone treatment compared to RF oxygen plasma is associated with the greater thickness of the modified layer [88,89].

Ma et al. [85] produced microfluidic devices with standard soft lithography techniques. The effects of curing time on surface modification by UV-ozone was presented where thermal curing eliminates low molecular weight species in PDMS, reducing hydrophobic recovery post plasma treatment. Additional curing time accelerates the surface hydrophilisation of PDMS.

**Figure 9:**
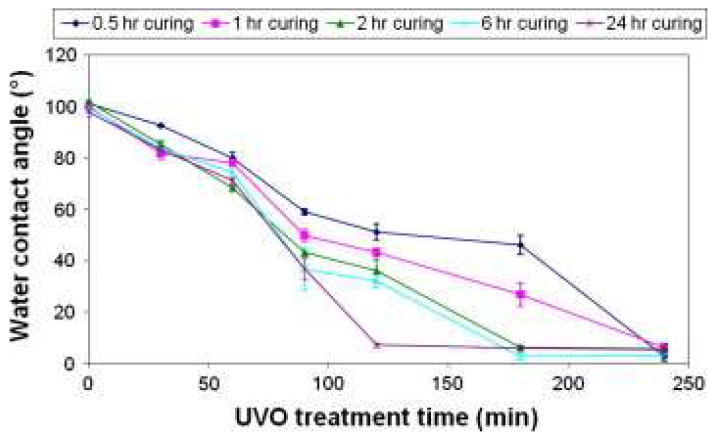
The effect of curing time on the surface hydrophilisation process of PDMS at 80 °C. Obtained from [85].

According to Figure 9, for a fixed curing temperature of 80 °C, in the first 60 min after UV-ozone treatment, there was a small discrepancy in the WCA for PDMS cured at different times, except for 60 min. However, notable differences emerged after 1 h of UV-ozone exposure, indicating that PDMS pieces with longer curing times show faster surface hydrophilisation. The maintenance of wettability by immersion in water was also analysed, where initially there was a reduction in the WCA with water for all PDMS parts, suggesting water absorption by the PDMS matrix.

**Figure 10:**
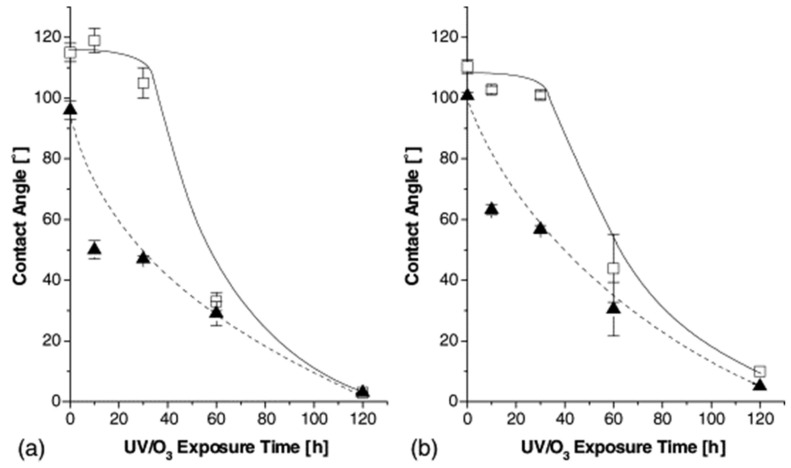
Water contact angles as a function of UV-ozone exposure time. (**a**) Sylgard 184, and (**b**) Sylgard 170. (□) Advancing and (▴) receding contact angles. The error bars indicate the standard deviation, and the dashed and solid lines serve to guide the eye only. Obtained from [90].

**Figure 11:**
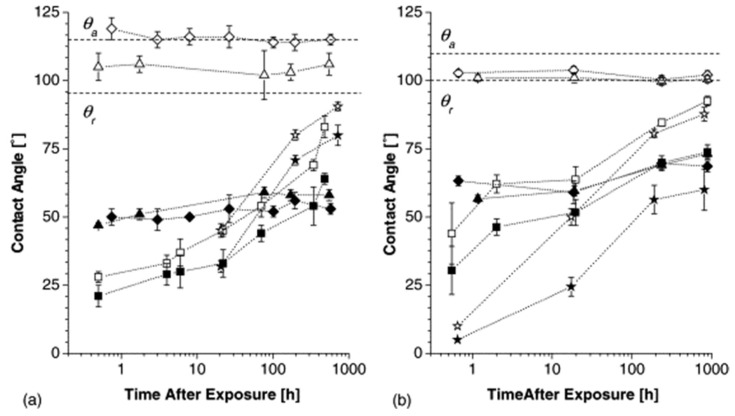
Hydrophobic recovery of PDMS after 10–120 min exposure to UV-ozone: (**a**) Sylgard 184, (**b**) Sylgard 170. The dotted lines indicate the values of the initial advancing (θa) and receding contact angles (θr). Empty symbols: advancing contact angles, filled symbols: receding contact angles. Diamond: 10 min exposure; triangle: 30 min exposure; square: 60 min exposure. The horizontal, dashed lines indicate advancing and receding contact angles, respectively, of untreated PDMS. Obtained from [90].

Oláh et al. [90] carried out a study focusing on the Johnson–Kendall–Roberts (JKR) contact mechanics methodology to investigate PDMS samples before and after surface treatment with UV-ozone. With increasing exposure to UV-ozone, the gradual formation of a hydrophilic surface layer similar to silica was observed below 20° WCA (Figure 10). Subsequently, there was a hydrophobic recovery evidenced by the increase in WCA (Figure 11). This phenomenon supports the hypothesis that hydrophobic recovery results, mainly from the progressive coverage of a permanent silica-like structure with free siloxanes and/or reorientation of polar groups.

The same study [90] showed that on PDMS with homogeneously dispersed filler (Sylgard 184), the surface roughness decreased as the oxidised region “collapsed” to form a smooth SiOx layer (final roughness < 2 nm). On the other hand, on PDMS with heterogeneously aggregated filler particles (Sylgard 170), the surface roughness increased with the treatment dose due to the “collapse” of the oxidised region, exposing the contours of the underlying filler aggregates (final roughness ≈140 nm). The effect of UV-ozone treatment on surface roughness varied between Sylgard 184 and 170, due to differences in filler content and type. Atomic force microscopy revealed that Sylgard 184 has smooth, homogeneous surfaces, with the root mean square of the roughness decreasing from 4.0 ± 0.4 to 1.9 ± 0.2 nm after exposure of 20 × 20 μm^2^. This pattern is consistent with previous observations by Vasilets et al. [41] on PDMS exposed to UV-ozone. On the other hand, Sylgard 170 exhibited a rougher initial morphology, with a roughness of 24 ± 1.2 nm, and roughness values increasing with exposure time, probably due to the higher filler content [90].

## 4. Surfactant Addition

Several wettability modification methods have limitations, such as restricted chemical stability and complexity in microfluidic channels [91]. Problems such as surface cracking, increased roughness, and loss of elasticity occur in some modifications, limiting the usefulness of PDMS surfaces [92,93]. To overcome these challenges, one of the possibilities is to use silicone-based molecules as direct functionalisation agents, preserving the natural characteristics of elastomers. Wetting agents, surfactants that usually consist of hydrophobic and hydrophilic portions, are widely recognised as useful substances for facilitating the dispersion of aqueous solutions on surfaces that are naturally hydrophobic [94,95]. The main surface modification methods analysed in this section are modification by bulk mixing and immersion in a solution.

### 4.1. Effects of Surface Coating Treatment with Surfactants on PDMS

Fatona et al. [91] developed an innovative approach to functionalise PDMS in a single step, using standardised moulds to define the surface areas to be modified, with functionalisation taking place during the vulcanisation of the PDMS. This method allowed for the creation of matrices in which ionic and non-ionic surfactants were applied to the surface of the elastomer during the curing process. This resulted in the spatial organisation of charged and uncharged alkyl/polymer chains in the PDMS.

In their experiment, seven tested surfactants led to hydrophilic surfaces after one-step mould modification, with significantly lower WCA than unmodified PDMS (109°). The stability of the hydrophilicity of the modified surfaces and the intensity of the interactions between the PDMS and the surfactants were evaluated by immersing the treated PDMS surfaces in high-purity water (18.2 MΩ) for 20 h. It was observed that, in most cases, immersion of the treated surfaces resulted in a significant change in the WCA. As expected, the interactions between the PDMS and the ionic surfactants proved to be weak, since these surfactants were solvated when the modified surfaces came in contact with water.

The influence of temperature on the hydrophilicity of the triblock copolymer and PDMS surfaces treated with Silsurf was also examined. After treatment, the modified surfaces were immersed in water for two hours, dried with nitrogen, and then incubated at different temperatures for one hour before the surface WCA was evaluated [91].

Table 2 represents a summary of the changes in WCA as a function of the addition of the seven surfactants, such as WCA measurements of surfactant functionalised PDMS surfaces; measurements performed on PDMS surfaces as prepared before soaking, after soaking, and after 11 days of storage in air; quantification of WCA measurements for all surfactant-modified PDMS surfaces; and quantification of WCA measurements for surfactant-modified PDMS surfaces at different temperatures, performed in the study mentioned above.

Based on the results provided in Table 2, it can be seen that the morphology of the treated surfaces varied, including opaque and roughened surfaces, smooth surfaces with depressions of various sizes, and surfaces with small dimples. Tween 20 exhibited complete WCA reversal post-soaking and had the smoothest and clearest surfaces with the lowest roughness. Silsurf A008-UP showed complete WCA reversal post-soaking and optically clear surfaces with small dimples. Alkyl (o-Wet)-treated surfaces had micrometre-wide depressions, while siloxane (n-Wet)-treated surfaces had sub-micrometre-wide depressions. Siloxane (a-Wet) demonstrated a post-soaking WCA change and displayed a specific morphology with more highly branched siloxane.

**Figure 12:**
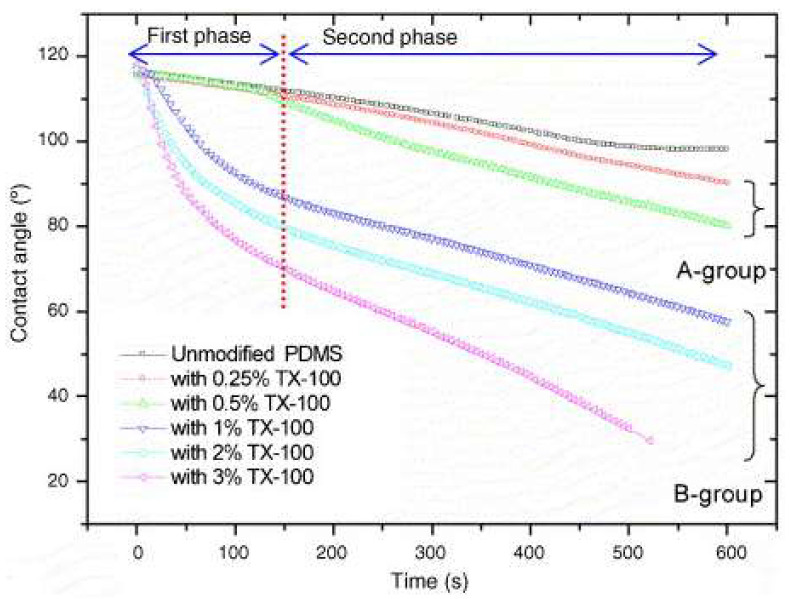
Dynamic contact angle measurements on six different modified PDMS surfaces; the contact angle change shows two different phases consisting of a large initial change and a subsequent gradual change. Adapted from [96].

Seo et al. [96] modified the surface of PDMS using the non-ionic surfactant Triton X- 100, varying its concentration, making it hydrophilic. As observed in Figure 12, in the PDMS groups modified with more than 1% TX-100 (group B), the WCA began to decrease in a matter of seconds. On the other hand, in the PDMS group with a concentration of less than 0.5% (group A), there was a delay before the change in WCA began. Compared to the gradual change in the WCA of the unmodified PDMS, the modified PDMS showed a sudden change, directly proportional to the concentration of TX-100 in the material. For example, within 150 s, the WCA decreased to 70° on PDMS with 3% TX-100.

**Figure 13:**
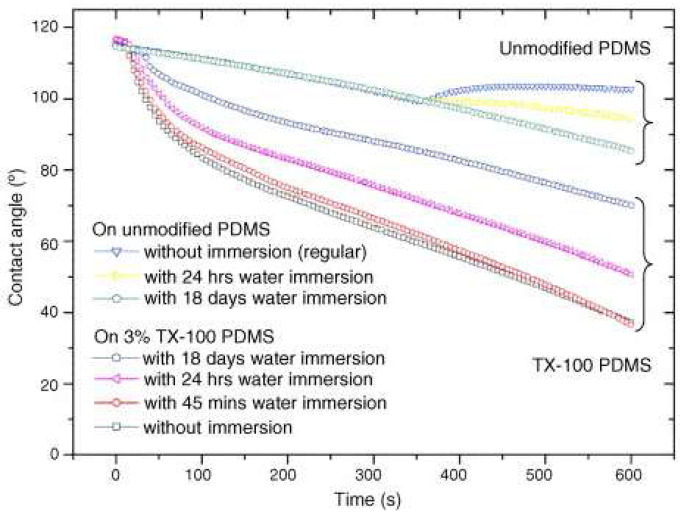
Contact angle measurement before and after immersion. The contact angles were measured right after immersion. For 45 min to 18 days of immersion in water, the wettability of PDMS decreased. Surfactant depletion by solvent immersion is a useful technique to control the wettability of the modified PDMS. Adapted from [96].

Immersed pairs of 3% PDMS TX-100 and unmodified PDMS substrates in deionised water for 45 min, 24 h, and 18 days shown are in Figure 13. WCA measurements were made after each surface was dried with dry nitrogen gas for 2 min after immersion.

The results indicated that the accumulation of surfactant at the interface between the modified PDMS and a drop of water caused a substantial change in the WCA in the initial phase. In addition, the reduction of surfactant through immersion in solvent represents a second strategy to control the wettability of the modified PDMS, complementing the initial control of the TX-100 concentration in the PDMS. In addition, it was shown that the WCA after 7 days did not show much difference, proving good durability [96].

Nam and Yoon [97] performed an experiment using surface/bulk treatment separately and also together, with the formation of a 3D interconnected pore network and the addition of a biocompatible surfactant (Silwet L-77). Porous PDMS (p-PDMS) was made into a 3D interconnected pore network with different pore sizes of 92.15, 176.78, 355.45, 634.40, and no pore (μm). The surfactant-added PDMS (s-PDMS) in which Silwet L-77 was added in different concentrations of 0.0, 0.1, 0.5, 1.0, 2.0, 4.0, and 8.0% by weight, and porous PDMS with added surfactant (ps-PDMS) with various pore sizes from 92.15 to no pores (μm), with various concentrations of Silwet L-77 from 0.0 to 8.0% by weight.

The results presented in this study reveal that the higher the percentage of Silwet L-77 surfactant, the lower the WCA. It was also noted that the smaller the pore sizes, the more hydrophobic the material became. There was a combined effect on the bonding of the two.

**Figure 14:**
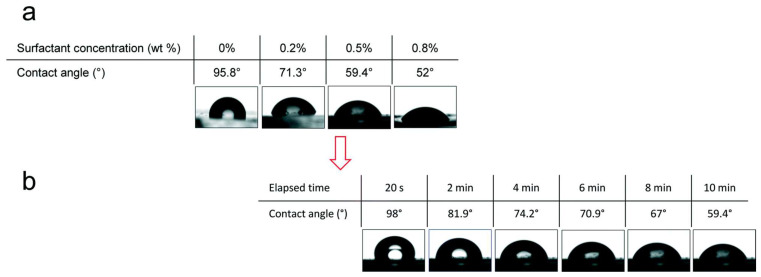
Contact angles of the water droplets on the surface of surfactant-added PDMS. (**a**) Wettability profile of poly(dimethylsiloxane) (PDMS) with different concentrations of surfactant (Silwet L-77) after 10 min. (**b**) Kinetics of PDMS–Silwet L-77 (0.5%) wettability during 10 min. Obtained from [98].

Another study with the surfactant Silwet L-77 by Montazeri et al. [98] where the concentration of 0, 0.2, 0.5, and 0.8% by weight of surfactant was altered, also by modification by Bulk mixing, showed a decrease in the WCA as the concentration of Silwet L-77 increased, as shown in Figure 14.

**Figure 15:**
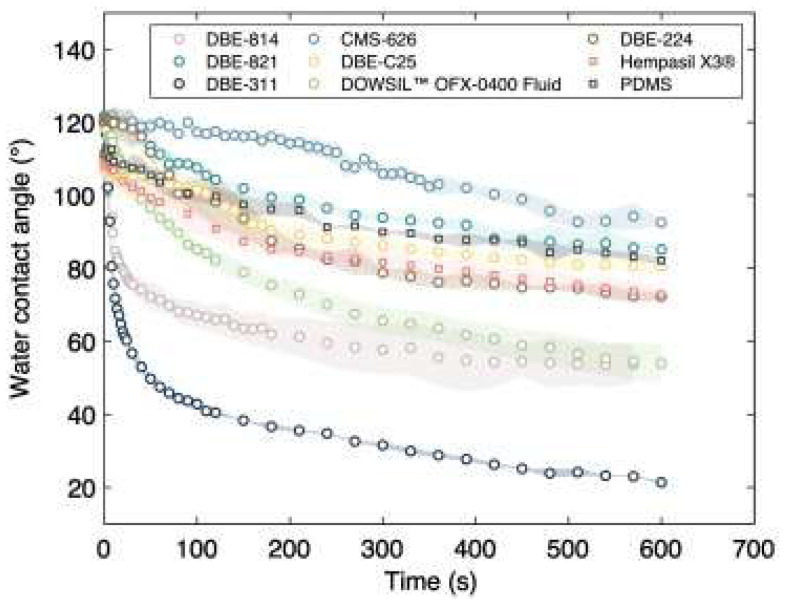
Kinetics of water contact angle (θw) with time for each surfactant-based PDMS coating and for surfactant-free PDMS coating, using 120 μm thickness coatings. The shaded area represents the standard deviation of contact angle measurements. Obtained from [99].

Soriano-Jerez et al. [99] selected seven different commercial PDMS-PEG copolymer non-ionic surfactants, namely, CMS-626, DBE-224, DBE-311, DBE-814, DBE-821, DBE-C25, and DOWSIL™ OFX-0400. The coatings were prepared by mechanically mixing Sylgard 184™ (base: curing agent 10:1 *w*/*w*) with 4% surfactant by weight. The WCA was measured for 10 min, as shown in Figure 15. Their results confirmed other studies, previously presented, where the WCA decreased over time, making the surface hydrophilic.

The most effective coatings, such as those based on DBE-311 and DBE-814, showed a rapid change in the WCA over time, indicating a low adsorption of bovine serum albumin (BSA). This was also observed to a lesser degree in the DOWSIL™ OFX-0400 fluid and DBE-224-based PDMS coatings. High-performance surfactants tend to have shorter induction times, associated with a faster reduction in surface tension. Molecules with smaller surface areas and branched-chain surfactants showed a faster reduction in surface tension. Surfactants such as DBE-821 or CMS-626, with a high PEG content, should also have short induction times, but this was not evident in the WCA measurements.

Table 3 summarises the interactions between various surfactants and PDMS surfaces by categorising surfactants by their binding type (ionic or non-ionic), estimating bond strength and stability. The table highlights the prevalence of non-ionic surfactants interacting with PDMS through a combination of hydrogen bonding and hydrophobic interactions. While bond strength and stability are generally classified as moderate and medium-term for most entries, specific details regarding these properties require further investigation for several surfactants. Overall, the table provides a valuable overview of surfactant–PDMS interactions, but further studies are needed to explore the detailed binding mechanisms, the impact of surfactant structure, and the long-term stability of these modified surfaces. Additionally, application-specific optimisation remains an area for future research to tailor surfactant selection for specific functionalities.

### 4.2. Applications of Surfactant-Treated PDMS

The in-mould functionalisation strategy is used to produce self-driven microfluidic devices with stable flow rates, adjustable by the geometry of the device. The in-mould method has potential for various surface modifications in applications such as analytical separations, biosensing, cell isolation, and small molecule discovery [91]. Likewise, it is attractive in micro/nano biomedical applications [96,107,108].

It has been observed that wettability depends on the chemical structure of the surfactants and their concentration. A disadvantage of using a wetting agent to improve wettability is the potential to undesirably influence the composition of the solution. Therefore, very low concentrations of the wetting agent are often used, which can be complicated when limited quantities of the target solution are available. In this context, the controlled and gradual release of a wetting agent from the base material of a micro/nano device is preferable to the direct addition to the solution [109,110].

## 5. Incorporation of Nanomaterials

There are several reasons for using nanomaterials to modify the wettability of PDMS. Firstly, the intrinsic hydrophobic characteristic of PDMS, stemming from its siloxane structure, can restrict its application in certain contexts that require hydrophilic surfaces. A versatile approach to overcoming these limitations is offered by the incorporation of nanomaterials, which allows the wettability properties to be tailored to the specific needs of the application [111,112].

When added to PDMS, nanomaterials can considerably modify the interactions at the solid–liquid interface, which directly affects the wettability of the surface. Specific characteristics, such as controlled roughness, can be caused by the nanotopography resulting from the presence of these materials and affecting the interaction between PDMS and liquids. These adjustments to the surface morphology can cause alterations to the contact angle and therefore to the wettability in its entirety [69,113,114].

The underlying principles in this wettability modification include the interaction between the specific properties introduced by the nanocomponents and the PDMS surface. The presence of these factors in the stability and durability of wettability changes caused by nanomaterials is attested to in the scientific literature [30,44,45,46].

This session aims to provide an understanding of the results obtained in research where nanomaterials have been used to alter the wettability of the PDMS surface by examining the most recent studies in this field.

The work of Wen et al. [115] addressed the production of fluorine-free superhydrophobic coatings applied to cotton fabrics. This process involved the use of silver nanoparticles (Ag), combined with the graft polymerisation technique and the application of PDMS to the fabric. During the manufacture of the coating, the surface of the cotton fabric was initially grafted with polyglycidyl methacrylate (PGMA) and functionalised with diethylenetriamine (DETA). Subsequently, silver nanoparticles were immobilised, followed by coating with PDMS (5% by mass of PDMS in ethyl acetate), resulting in a superhydrophobic coating on the cotton fabric.

The coated cotton fabric showed a good superhydrophobic capacity, with a WCA of 155° ± 1.5°. This characteristic was achieved by effectively combining PDMS with silver nanoparticles (Ag), which played the role of hydrophobic agents, decreasing the surface energy of the fabric and promoting robust adhesion of the nanoparticles to increase surface roughness. In addition, the durability of the coating was assessed at different pH levels and through a weight abrasion method with more than 200 cycles. The results highlighted the remarkable strength and durability of the coating, maintaining superhydrophobicity with a WCA of over 150° in both acidic and alkaline environments [115].

**Figure 16:**
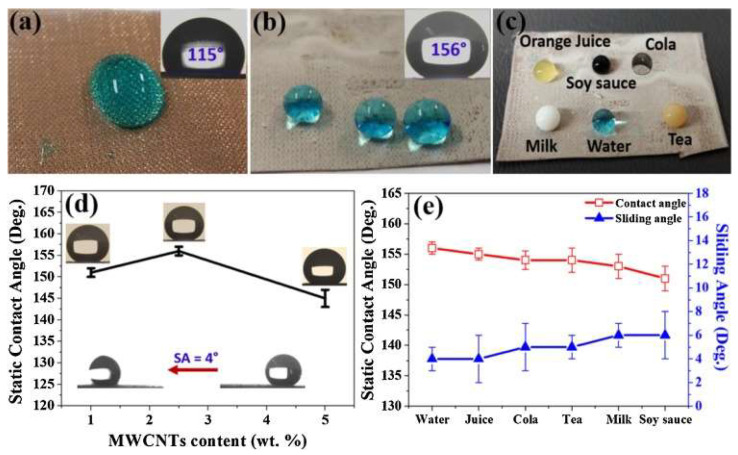
Pictures of water droplets on the (**a**) untreated and (**b**) superhydrophobic Cu mesh (modified with 2.5 wt.% composite coating), with corresponding WCAs. (**c**,**e**) The photo and WCAs of various liquid droplets on the coated mesh, respectively. (**d**) The change in the WCA of the prepared surfaces as a function of the content of MWCNTs in the composite coatings. Obtained from [116].

Barthwal et al. [116] developed fluorine-free superhydrophobic coatings on copper mesh using PDMS in conjunction with a multi-walled carbon nanotube/zinc oxide (MWCNTs/ZnO) composite, using dip-coating techniques. In this study, the sol–gel technique was used to synthesise the MWCNTs/ZnO composite. It is noteworthy that the mesh coated with 2.5% by weight of the MWCNTs/ZnO composite exhibited greater superhydrophobicity, with a WCA of 156° and a sliding angle of 4°, compared to the coatings containing 1% by weight (151°) and 5% by weight (145°) of the composite, as illustrated in Figure 16.

The superhydrophobicity manifested by the copper mesh is the result of the presence of hierarchical micro/nanostructures, giving the coated surface greater roughness. Additionally, it was observed that the PDMS-based coating on the copper mesh preserves its superhydrophobic characteristic in the face of various unfavourable environmental conditions. This includes extreme temperature variations; exposure to corrosive environments, such as a 3.5% by weight NaCl solution; and resistance to strongly acidic/alkaline solutions. This ability to maintain superhydrophobicity highlights the robustness and adaptability of the coating proposed by the research in question [116].

Sadler and Crick [117] developed an affordable and direct superhydrophobic filtration method by applying a PDMS coating to glass microfiber filters. This approach aimed to separate oils from water both through suction pressure and under the influence of gravity. The average WCA of the PDMS-coated filter was determined to be 158 ± 3°, in contrast to the 95° observed on the flat PDMS surface. This significant difference can be attributed to the rough morphology induced by the filters, combined with the presence of PDMS as a low-surface-energy agent.

## 6. Modification of PDMS Surface Wettability for Microfluidic Applications

Given the growing use of treatments to modify the wettability of the PDMS surface for microfluidic applications, additional successful studies are described below, addressing the different methods mentioned in this review. PDMS’s non-toxic, biocompatible, stable, and flexible properties make it a well-known material used in microfluidics. However, its inherent hydrophobicity presents challenges in fluid handling applications, as previously discussed. Various surface treatment methods have been studied to improve the wettability of PDMS, including gas-phase processing techniques such as oxygen plasma [118,119,120] and UV irradiation [31], as well as chemical methods such as LBL deposition [31] and others. Modification with surfactants has emerged as a promising approach to long-term hydrophilicity, offering simplicity and effectiveness without the need for complex procedures [121,122].

**Figure 17:**
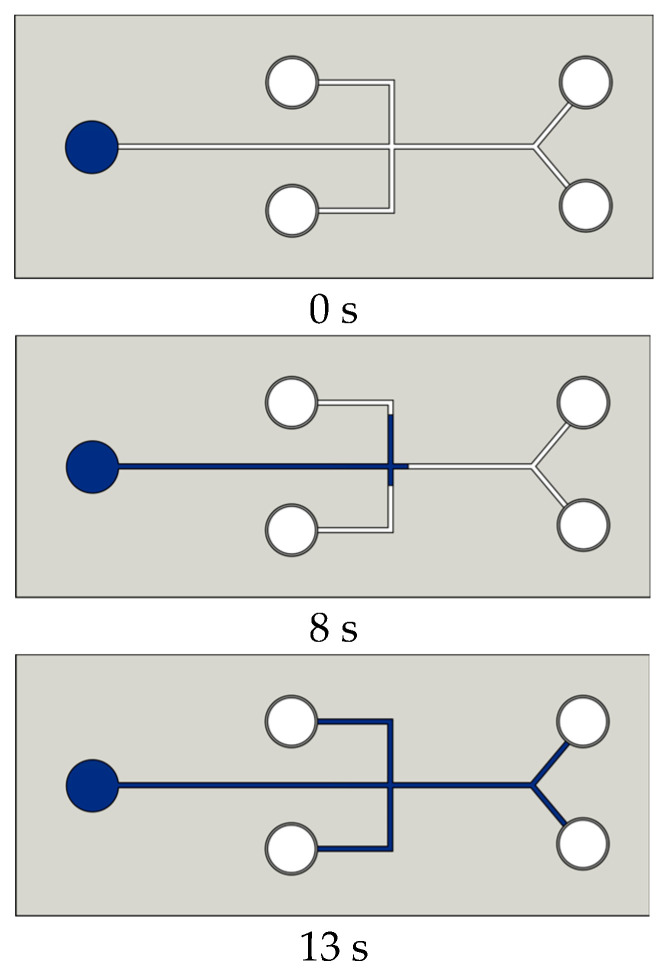
Schematic diagram of a capillary test on PDMS after 420 h of treatment by O_2_-plasma-PEG based on the work of Long et al. [120]. The images make it clear that at 8 s the Rhodamine B fluid was halfway through the channel and at 13 s the channels were completely filled. With untreated PDMS, no flow was observed at least during the first 60 s.

Long et al. [120] performed a surface modification of PDMS material by oxygen plasma, followed by PEG coating, for hydrophilic enhancement on pure PDMS. By using rhodamine droplets, it was tested in a capillary-driven microfluidic device. From Figure 17, it is clear that at 8 s, the Rhodamine B fluid was halfway through the channel, and at 13 s, the channels were completely filled. With untreated PDMS, no flow was observed at least during the first 60. This method has shown long-term hydrophilic surface modification as the fluid could flow without external pumping for a period of 420 h.

Peterson et al. [119] tested native and oxidised PDMS coatings (ox-PDMS) as biocompatible coatings for microfluidic devices. Glass-silicon microfluidic devices coated with hydrophilic ox-PDMS had an undisturbed flow rate over 14 min of operation, while the uncoated device suffered a loss in rate of 12%, and the native PDMS coating showed a loss of almost 40%.

By using surfactants to modify the surface of PDMS, Holczer et al. [123] carried out a controlled modification to develop an autonomous capillary-driven microfluidic system to be applied to bioanalytical devices. Vilčáková et al. [124] realised four types of CNT-based composites of various concentrations from 0 to 6% by volume of surfactants: anionic surfactant dodecylbenzene sulphonic acid (DBSA), cationic surfactant cetyltrimethylammonium bromide (CTAB), and a DBSA/CTAB surfactant mixture, which were prepared by simple mechanical mixing and sonication, leading to a homogeneous distribution of a filler in a silicone matrix.

Wu and Hjort [125] introduced a non-ionic surfactant, Pluronic F127, into the PDMS pre-polymer before curing. By filling the microchannel with water, the Pluronic F127 molecules incorporated into the PDMS migrated towards the water/PDMS interface to minimise surface energy. This phenomenon resulted in a hydrophobic interaction between the PPO and PDMS segments, causing the hydrophilic PEO segments to extend outwards from the surface. The CA of the PDMS surface modified by Pluronic F127 changed from 99 to 63° after the sample was immersed in water for 24 h, compared to a CA of 104° for the native PDMS.

Recently Gonçalves et al. [126] modified the PDMS surface properties by using three different surfactants, i.e., Pluronic^®^ F127, polyethylene glycol (PEG), and polyethylene oxide (PEO). In this study, they found that the bulk modification performed with the PEO surfactant at 2.5% was the most promising candidate to enhance blood plasma separation efficiency in microfluidic devices, as it facilitates the fluid flow, reduces cell aggregations and air bubble trapping, and achieves higher levels of sample purity.

## 7. Promising Trends and Future Prospects

After reviewing previous techniques, it can be seen that they had some limitations, such as the reduction in long-term efficacy for surfactant addition, the lack of precise control over plasma parameters for oxygen plasma treatment, non-uniformity, and lack of depth and degradation in the material to prolonged exposure to radiation in UV-ozone treatment. The challenges of guaranteeing uniform and stable dispersion of the nanomaterials in the PDMS matrix were also addressed, as well as concerns related to their toxicity and compatibility in biological applications. However, new techniques have emerged to overcome some of these shortcomings, as well as the combination of different methods to take advantage of the individual benefits of each one.

One of the emerging trends for modifying the surface of PDMS is surface nanotexturisation, which involves creating nanostructured patterns on the surface. These patterns can be achieved using techniques such as electron beam lithography, nanoimprint lithography, and nanoimprinting techniques [114,127]. Nanotexturisation can increase the surface area available for interactions with water, resulting in greater hydrophilicity, and it also provides greater durability, precise control over surface characteristics, and biological compatibility. However, this process is more costly for small-scale production, despite being interesting on an industrial scale [128].

Another promising approach is the deposition of thin films of nanomaterials, such as metal oxides [129] or conductive polymers [130], on the PDMS surface. These films can be deposited using techniques such as sputtering [131], chemical vapour deposition (CVD), [130] or dipping techniques [132]. This technique has better uniformity and compatibility with manufacturing processes as it is versatile and compatible with a variety of manufacturing processes, and also it has a wide range of materials, providing greater flexibility in the choice of materials to meet the specific demands of each application.

As work progresses, it is likely, based on current studies, that there will be greater integration of multiple PDMS surface modification techniques. This may involve combinations of the traditional techniques that were discussed throughout the article, as well as new techniques such as nanotexturisation and thin film deposition, to achieve optimal combinations and enhance the desired properties of the material, seeking not only the optimisation of hydrophilicity but also to control other important properties such as biocompatibility, chemical resistance, and durability. However, a major disadvantage of this approach, when compared to traditional methods of modifying wettability on the surface of PDMS, is the complexity and cost associated with the process.

## 8. Conclusions

This comprehensive review of the surface wettability of polydimethylsiloxane describes the importance and complexity of surface modification techniques for altering the inherent hydrophobicity of PDMS. The four main strategies, namely, oxygen plasma treatment, the addition of surfactants, UV-ozone treatment, and the incorporation of nanomaterials, are the traditional methods most used to modify the wettability of the PDMS surface due to the greater availability of information, having lower complexity compared to the new techniques and lower cost associated with them. This has highlighted the difference in methods that can be used to adapt PDMS surface properties to specific applications. Each of the techniques has its advantages and challenges. For example, oxygen plasma treatment has become an important method for increasing the hydrophilicity of the surface by introducing polar functional groups through oxidation reactions. The addition of surfactants, on the other hand, provides versatility for altering wettability, with the choice and concentration of the type and quantity of surfactant being decisive for achieving the desired surface properties. UV-ozone treatment stands out for its effectiveness in increasing surface energy, inducing oxidation and also generating hydrophilic functional groups. Finally, the incorporation of nanomaterials into PDMS matrices appears to be a promising technique for altering wettability, with the option of having adaptable surface properties through controlled distribution and interfacial interactions. In future works, the combination of these techniques has great potential to satisfy the future needs of various domains, as well as the use of new techniques that are emerging on the market, which will be increasingly explored and consequently have a lower cost and less complexity.

## Figures and Tables

**Table 1:** Some advantages and disadvantages of different methods to fabricate PDMS-based superhydrophilic/superhydrophobic coatings.

Methods	WCA (and Over Time) ≈	Advantages	Disadvantages	Applications	Ref.
Oxygen plasma treatment	17°–46° (50°–115°, after 6 h) *	Low cost, ease of implementation, and compatibility with sensitive materials	Fast hydrophobic recovery and limitations on penetration depth	Microfluidic devices	[39]
Oxygen plasma treatment	40°–101, 17° *			Improvement of polydimethylsiloxane (PDMS) surface biocompatibility as the most used biomaterial in maxillofacial prostheses for intraoral defects	[33]
Oxygen plasma treatment (SRMJ)	70°	Biological cells adhered more easily to surfaces		Controlling biological cells’ attachment on biocompatible polymer material	[40]
UV-ozone treatment	10°–40° (40°–95°, after 30 days) *	Quick process and room temperature operation	Temporary surface modification and potential for degradation	Microfluidic	[41]
UV-ozone treatment	7°, after 240 min UV/O treatment				[42]
Surfactant addition	18°–68° (Table 2)	Ease of application; immediate effect and versatility	Uniformity challenges and potential leaching	Microfluidic, microfluidic biomedical, and non-toxic antibiofouling coatings	[43]
Incorporation of nanomaterials	<150°	Long-lasting modification and improvement of mechanical properties	Dispersion challenges, ecological problems, and high costs	Antibacterial activity and oil–water separation	[44]
Incorporation of nanomaterials	<150°			Self-cleaning, oil–water separation, and flame-retardant properties	[45]
Incorporation of nanomaterials	158°			Oil–water separation	[46]

* Varies depending on the time of PDMS exposure to the treatment.

**Table 2:** Representation of the wettability of PDMS surfaces functionalised with each surfactant molecule, based on the work of Fatona et al. [91].

Surfactant	WCA (Before Soaking)	WCA (After Soaking)	WCA (After 11 Days or More)	WCA (21 °C)	WCA (60 °C)	WCA (80 °C)	WCA (100 °C)	Chemical Structure	Morphology before Soaking
Unmodified PDMS	109°	-	-	-	-	-	-	(-Si(CH_3_)_2_-O-)n	-
Sodium dodecyl sulphate (SDS)	57°	114°	-	-	-	-	-	CH_3_(CH_2_)_11_OSO_3_Na	Opaque and roughened surfaces
Cetyl trimethylammonium bromide (CTAB)	38°	106°	-	-	-	-	-	C_16_H_33_N(CH_3_)_3_Br	Opaque and roughened surfaces
Tween 20	39°	91°	Complete reversal	-	-	-	-	CH_3_(CH_2_)_18_(OCH_2_CH_2_)nOH	Optically clear and lowest surface roughness
Silsurf A008-UP	20°	63°	Complete reversal	≈40°	≈49°	≈67°	≈68°	-	Optically clear and surfaces presenting small dimples
* Alkyl (o-Wet)	43°	36°	-	≈40°	≈48°	≈53°	≈68°	(PEG) − (PDMS) − (PEG) − (Alkyl)	Smooth surfaces with depressions (microns wide)
* Siloxane (n-Wet)	47°	27°	-	≈38°	≈36°	≈38°	≈40°	(PEG) − (PDMS) − (PEG) − (Si-O)	Smooth surfaces with depressions (sub-micron wide)
* Siloxane (a-Wet) {has a more highly branched siloxane}	22°	25°	40°	≈21°	≈18°	≈19°	≈19°	(PEG)−(PDMS)− (PEG)−(Si-O) branched	-

* Poly(ethyleneglycol)-silicone-poly(ethyleneglycol) (PEG-PDMS-PEG) non-ionic triblock copolymers with terminal functionalities.

**Table 3:** Surfactant–PDMS interactions: binding type, strength, and stability.

Surfactant	Binding Type	Bond Strength	Bond Stability	Notes	References
Sodium dodecyl sulphate (SDS)	Ionic	Strong	Long-term	Bond strength and stability may vary depending on concentration and chain length.	[100]
Cetyl trimethylammonium bromide (CTAB)	Ionic (similar to SDS)	Strong	Long-term	Specific information on binding type, strength, and stability may require further research or data from the supplier.	[101]
Tween 20	Non-ionic (hydrogen bonding, hydrophobic interactions)	Moderate	Medium-term	Specific information on binding type, strength, and stability may vary depending on the specific alkyl surfactant.	[102]
Silsurf A008-UP	Non-ionic (likely hydrogen bonding, hydrophobic interactions)	Moderate	Medium-term	Specific information on binding type, strength, and stability may vary depending on the specific siloxane surfactant.	[103]
* Alkyl (o-Wet)	Non-ionic (hydrophobic interactions)	Moderate	Medium-term	Specific information on binding type, strength, and stability may vary depending on the specific siloxane surfactant.	[104]
* Siloxane (n-Wet)	Non-ionic (hydrophobic interactions, Van der Waals forces)	Moderate	Medium-term	Bond strength and stability may vary depending on concentration and temperature.	[105]
Siloxane (a-Wet)	Non-ionic (hydrophobic interactions, Van der Waals forces)	Moderate	Medium-term	Specific information on binding type, strength, and stability may require further research.	[106]
Triton X-100	Non-ionic (hydrogen bonding, hydrophobic interactions)	Moderate	Medium-term	Specific information on binding type, strength, and stability may require further research.	[106]
Silwet L-77	Non-ionic (hydrophobic interactions, Van der Waals forces)	Moderate	Medium-term	Specific information on binding type, strength, and stability may require further research.	[98]
CMS-626	Non-ionic (likely hydrogen bonding, hydrophobic interactions)	Moderate	Medium-term	Specific information on binding type, strength, and stability may require further research.	[99]
DBE-224	Non-ionic (likely hydrogen bonding, hydrophobic interactions)	Moderate	Medium-term	Specific information on binding type, strength, and stability may require further research.	[99]
DBE-311	Non-ionic (likely hydrogen bonding, hydrophobic interactions)	Moderate	Medium-term	Specific information on binding type, strength, and stability may require further research.	[99]
DBE-814	Non-ionic (likely hydrogen bonding, hydrophobic interactions)	Moderate	Medium-term	Specific information on binding type, strength, and stability may require further research.	[99]
DBE-821	Non-ionic (likely hydrogen bonding, hydrophobic interactions)	Moderate	Medium-term	Specific information on binding type, strength, and stability may require further research or data from the supplier.	[99]
DBE-C25	Non-ionic (likely hydrogen bonding, hydrophobic interactions)	Moderate	Medium-term	Specific information on binding type, strength, and stability may require further research.	[99]
DOWSIL™ OFX-0400	Non-ionic (likely hydrogen bonding, hydrophobic interactions)	Moderate	Medium-term	Specific information on binding type, strength, and stability may require further research or data from the supplier.	[99]

## Data Availability

Not applicable.

## Abbreviations

**Nomenclature**		*Abbreviation*	
PDMS	Polydimethylsiloxane	*θ*	Contact angle
UV	Ultraviolet		
L	Liquid		
V	Steam		
S	Solid		
O_2_	Oxygen		
SiOH	Silanol group		
SEM	Scanning electron microscopy		
XPS–X-ray	Photoelectron spectroscopy		
DC	Direct current		
AFM	Atomic force microscopy		
SRMJ	Scanning radical microjet		
WCA	Water contact angle		
CA	Contact angle		
ccm	Cubic centimeters per minute		
PEG	Polyethylene glycol		
BSA	Bovine serum albumin		
RF	Radio frequency		
JKR	Johnson–Kendall–Roberts		
